# Barriers to effective management of primary postpartum haemorrhage following in-hospital births in northwest Ethiopia: healthcare providers’ views using a qualitative approach

**DOI:** 10.1186/s12884-022-05071-6

**Published:** 2022-10-08

**Authors:** Tiruneh Bewket, Fooladi Ensieh, Plummer Virginia, McLelland Gayle

**Affiliations:** 1 School of Nursing and Midwifery, Monash University, Wellington Rd, 3800 Clayton, Melbourne, VIC Australia; 2 School of Nursing, University of Gondar, Gondar, Ethiopia; 3 School of Health, Federation University, Berwick, Melbourne, Australia

**Keywords:** Health Personnel, Hospital Birthing, Maternal Health Services, Postpartum Haemorrhage

## Abstract

**Background:**

Data showed that postpartum haemorrhage contributed to over 40% of in-hospital deaths of Ethiopian women. However, little is known about the barriers to effective management of primary postpartum haemorrhage. This study aims to explore the views and experiences of maternity healthcare professionals about the barriers to managing primary postpartum haemorrhage following in-hospital births in northwest Ethiopia using the ‘Three Delays’ model as a conceptual framework.

**Methods:**

A qualitative descriptive study was employed at two tertiary referral hospitals between December 2018 and May 2019. Forty-one maternal healthcare providers, including midwives, midwifery unit managers, and obstetricians, participated in this study. Individual face-to-face interviews, focus group discussions, and self-administered open-ended questionnaires were used to collect data. A framework analysis approach was used for the qualitative data analysis. Themes were identified based on the Three Delays model of ‘delay the decision to seek care’, ‘delay arrival at a health facility’, and ‘delay the provision of appropriate and quality care’.

**Results:**

Participants reported several modifiable issues when managing primary postpartum haemorrhage, and all were linked to a delay in receiving appropriate and quality care due to limited resources. Five sub-themes were identified: ‘workforce’, ‘communication issues between healthcare providers’, ‘systemic issues’, ‘education, training, and resourcing issues’, and ‘lack of identification and referral’.

**Conclusion:**

Maternal healthcare providers in these hospitals require training in managing a birthing emergency. In addition, the birth units need adequate supplies and continuous essential services.

**Supplementary Information:**

The online version contains supplementary material available at 10.1186/s12884-022-05071-6.

## Background

In 2015, United Nations member states agreed on the 17 Sustainable Development Goals (SDGs), aiming to improve the global future. The third SDG is ‘Health and well-being for all’ [1] and includes the target of reducing the worldwide maternal mortality rate (MMR) to under 70 per 100,000 live births by 2030 [2]. Following the release of the SDGs, the World Health Organization (WHO) launched the Global Strategy for Women’s, Children’s, and Adolescents’ Health. Essentially, this aimed to reduce MMR using multiple strategies, including family planning, antenatal care, clean and safe birth services, and postnatal care [2]. Accordingly, since 2016, low-resource countries in Africa and Asia, including Ethiopia, have implemented changes to meet this target [3]. Yet, in 2017, 86% of the estimated global number of maternal deaths continued to occur in Africa and South Asian countries [4]. Despite these deaths, a 2.9% reduction in MMR was achieved worldwide in the same year [4].

Primary postpartum haemorrhage (PPPH) is a leading cause of maternal mortality, yet there continues to be a debate about its definition [5]. Globally, the most accepted definition is blood loss from the genital tract, within 24 h, of greater than 500 ml or 1000 ml from vaginal and caesarean birth, respectively [5].

In the broader literature, causes of PPPH are traditionally categorised according to the framework ‘4Ts.’ The 4Ts refer to ‘tone’ (weak uterine contraction), ‘trauma’ (genital tract injury), ‘tissue’ (retained products of conception), and ‘thrombin’ (coagulation disorders) [6]. Tone accounts for the majority of PPPH cases (80%), followed by trauma (13%), tissue (5%), and thrombin (2%) [7].

The immediate postpartum period is an exceptionally high-risk time for the death of women due to excessive bleeding. This is further aggravated in low-resource countries because of poor access to comprehensive maternity healthcare services [8], which have medical resources, including medications, blood, and staff necessary for the effective management of emergency situations [9].

Although the number of skilled maternity care practitioners in Ethiopia increased by 50% between 2016 and 2019 [10], the number of womens’ deaths following in-hospital births continues to be high [11]. Nearly three-quarters (71.7%, n = 725) of the 1010 maternal deaths occurred in healthcare facilities. Of these, more than nine out of ten (n = 658) were in hospitals, and the remaining (n = 67) died in health centres [11]. Similar to the global figures, the leading cause of maternal death in Ethiopia is both primary and secondary postpartum haemorrhage [11].

As recommended by the SDGs framework, contextualising the priorities of local women’s health needs and understanding local circumstances to facilitate improved healthcare systems requires the collection of high-quality data [2]. There is limited information regarding any potential system issues impacting the management of PPPH following in-hospital births in Ethiopia.

## Methods

Using the ‘Three Delays’ model as a conceptual framework [12], this study aims to explore the views and experiences of maternity healthcare professionals about barriers to managing PPPH following in-hospital births. The study was conducted in the Amhara National Regional State of Ethiopia from December 2018 to May 2019. The region, is one of nine national regional states. According to population projection, during 2019, the region has 22% of the Ethiopian population [13]. The Amhara National Regional State has the highest poverty rate, at 26%, compared to the national average of 24% [14]. Although data on maternal mortality are not available in this province, maternal health care services have improved over recent years. The skilled staff providing antenatal care have increased from 67% in 2016 to 83% in 2019. Skilled clinicians in birth services have increased from 10% in 2011 to 56% in 2019. Between 2014 and 2019, institutional birth services for women increased from 12–48% [15]. This study was conducted at the two tertiary referral hospitals, Felege Hiwot Referral Hospital and the University of Gondar Comprehensive Specialized Hospital. Combined, these hospitals provide the healthcare needs for over 12 million people, including antenatal care, birthing service, and postnatal care, with approximately 16,000 births in 2019 [16, 17].

This was a qualitative descriptive study design conducted among maternity healthcare professionals exploring the views and experiences about the barriers during the management of PPPH following in-hospital births. The study inclusion criteria were clinicians aged 18 and older who were midwives, midwifery unit managers (MUMs), and obstetricians working as permanent staff in the maternity unit for at least six months in the two tertiary referral hospitals in all categories. Participants on any leave at the time of data collection were excluded from the study.

Individual face-to-face interviews, focus group discussions (FGDs), and open-ended questionnaires were used to collect data from obstetricians, MUMs, and midwives, respectively. The interviews and FGDs (with 6–8 people in each group) were conducted in the ‘Amharic’ language to ensure the participants could express themselves freely without the constraints of a second language. We conducted four FGDs and four individual interviews that lasted 20–30 min each. Two facilitators, the first author and a research assistant, conducted the FGDs, while the first author conducted the interviews. The interviews were held in the MUM’s office, while the FGDs were conducted in the hospital conference room. Data collection continued until no new ideas emerged in the interviews or FGDs. Due to their different work commitments, an open-ended questionnaire in English was used to collect data from five obstetricians. The data collection instrument has a structured interview guide used during the interviews and FGDs (Supplement A).

All participants were given assurances of confidentiality. They signed an informed consent form, which was collected before participating in this study. Pseudonyms were used in published works. Moreover, during the data collection, the author did not collect any sociodemographic characteristics, such as age, service year, or income of the participants, to avoid being potentially identified. All the interviews and FGDs were recorded and transcribed verbatim for analysis.

The data collection instrument for this study was adopted from Timmings et al. [18]. The instrument was previously trialled in Ethiopia to understand barriers and facilitators to implementing guidelines (WHO) to prevent and treat postpartum haemorrhage [18]. The context and topic were similar to the current study. Furthermore, this pre-existing instrument was carefully designed to raise issues about barriers to managing postpartum haemorrhage at different levels: challenges at the system level; at the healthcare provider level; and at the women and community level, and thus, aligns with the conceptual framework of this study—the Three Delays model [12]. This data collection instrument has six predetermined questions for the interviews, FGDs, and open-ended questionnaires. The study participants were asked about their experiences, the activities they perceived to have been done well, and the support they received during the management of PPPH. The participants were also asked about team communication, particularly while managing PPPH. In addition, the participants were asked about challenges to the management of PPPH. Finally, the participants were asked if they could provide any additional information about managing PPPH.

Data were recorded digitally during the FGDs and interviews and then transcribed in the Amharic language. To maintain the data quality, transcripts were cross-checked against the recorded data daily. The dataset was then translated into English by another individual, who was able to speak both Amharic and English languages, as recommended by Abfalter et al. [19]. Several strategies were undertaken to ensure the rigour and trustworthiness of the study’s findings, including credibility, dependability, confirmability, and transferability [20]. To attain dependability, a coding and recoding technique was used as recommended by Lincoln and Guba [20]. Detailed quotes with disconfirming evidence were developed from the study participants’ information [21], and regular team meetings were held to discuss the identifying sub-themes and themes to enhance confirmability [22]. The author provided a detailed description of the research setting, sample and sample size, sampling strategy, the responsibility of study participants, inclusion criteria, and data collection techniques to assist the transferability of the findings [23].

In addition, the authors used the following methods to ensure the current study’s rigour and trustworthiness. The author built a trusting relationship with the invited study participants during the data collection period, which provided nuanced data about the PPPH management situation in these tertiary referral hospitals. The inclusion criteria for maternal healthcare providers were carefully designed and limited to permanent employees with more than six months of work experience at the study hospital. As a result, participants were more likely to openly describe their experience caring for a woman with PPPH after in-hospital childbirths. Furthermore, the interviews and focus groups were held in the local language, allowing participants to express themselves freely. This aided in collecting reliable data that represent issues encountered during the management of PPPH in northwest Ethiopia.

In this study, the data analysis was conducted using a framework analysis approach, according to Srivastava and Thomson in five steps, including ‘familiarisation’, ‘identifying’, ‘indexing’, ‘arranging’, and ‘interpreting’ [24]. The first phase occurs during the interviews, FGDs, and data transcription, and also during the further reading of the transcripts. The ultimate goal of this stage is to immerse the author in the specifics of each transcript to understand the entire dataset before breaking it down into parts and recognising common themes [25]. After familiarisation, the author identifies themes from the data. In Phase two, the recurrent themes were placed in a Microsoft Word Table (29). During the third phase of the data analysis, themes and sub-themes were refined, merged, and developed using framework analysis, levelling the identified sections of data to each theme [24]. The fourth phase arranged the themes in a chart. The final phase was the interpretation of the key characteristics of the themes [24]. Themes were identified based on the Three Delays model of ‘delay the decision to seek care’, ‘delay arrival at a health facility’, and ‘delay the provision of appropriate and quality care.’

## Findings

**Fig. 1:**
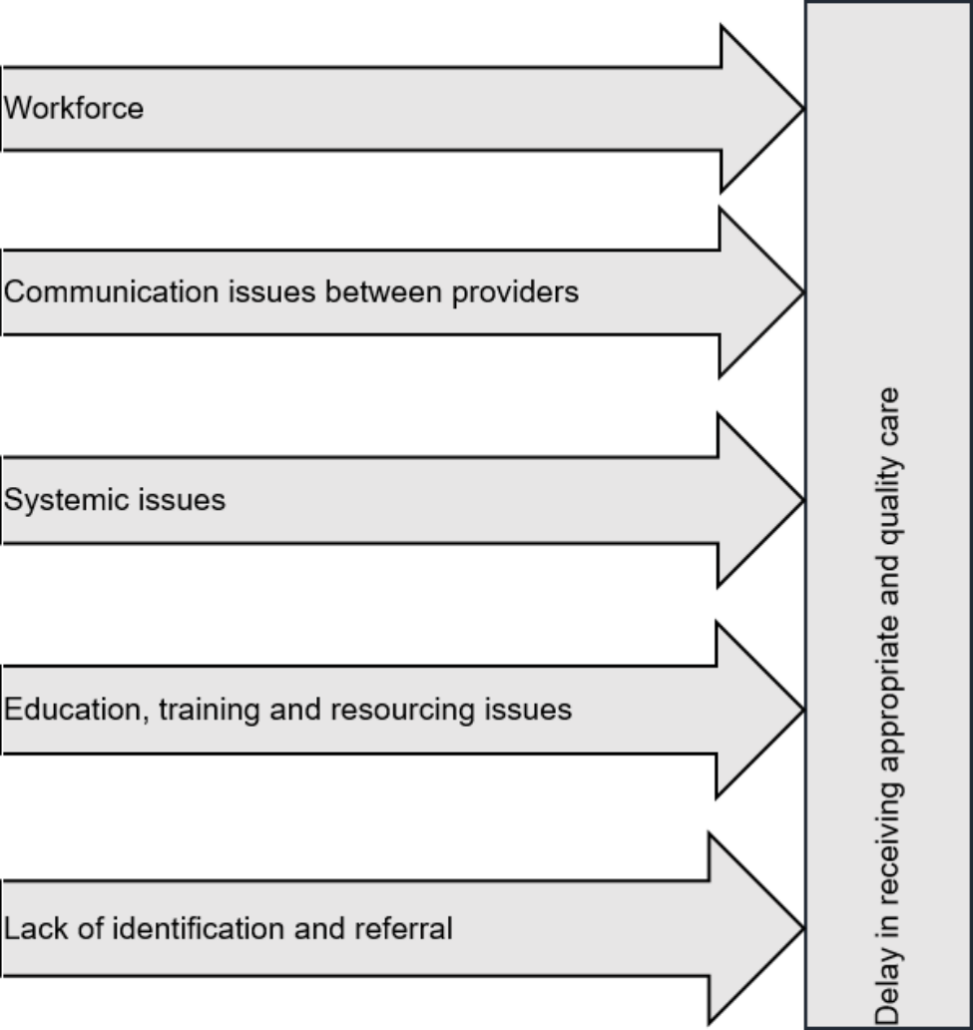
Barriers towards PPPH management following in-hospital births as perceived by caregivers in northwest Ethiopia (2018)

Overall, 41 maternal healthcare providers participated in this study, including 32 (78%) midwives, four (9.8%) MUMs, and five (12%) obstetricians. The midwives’ work experiences ranged from seven months to 13 years, while the MUMs’ work experience varied from three to 11 years. The obstetricians had between three and five years of work experience. The findings from the different groups of maternal healthcare providers showed several issues that particularly relate to the ‘delay in receiving appropriate and quality care’ or the third delay described by Thaddeus and Maine (1994). [12]. Figure 1 shows the major theme and sub-themes identified as barriers to managing PPPH following in-hospital births in northwest Ethiopia.

### Delay in receiving appropriate and Quality Care

Maternal healthcare providers reported several challenges in providing and receiving immediate care for women with PPPH in this study. Under the central theme of ‘delay in receiving appropriate and quality care’, five sub-themes were identified, including ‘workforce’, ‘communication issues between healthcare providers’, ‘systemic issues’, ‘education training’ and resourcing issues’, and ‘lack of identification and referral’.

#### Workforce (heavy workload and demotivated staff)

Most midwives claimed they became demotivated when they did not manage the complications associated with childbirth due to other hospital staff underestimating their midwifery knowledge and skills. During an FGD, one midwife identified that:

‘… there was an immense undermining view (underestimation of knowledge and skill) of the midwives …’ (Helen, Midwife).

In different FGDs, participants confirmed that they felt other hospital staff regarded midwives as less competent clinicians. The midwives explained there was a lack of midwifery job descriptions in the hospitals, which prevented them from using their expertise when supporting women with PPPH. As one participant noted:

‘… nowadays, there is no trust in midwives’ skills. The PPPH is … managed by medical interns. Due to the lack of job description, midwives have a hard time applying the full skill … (during the management of women with PPPH).’ (Sibhat, Midwife).

One MUM explained that midwives were not involved in caring for women with birth complications. Midwives were frequently directed to focus on technical skills, including intravenous securing, preparing, and transferring the required equipment to the medical doctor, rather than providing direct care to women with PPPH. Consequently, many midwives experienced demotivation and loss of morale when working. One MUM stated:

‘Midwives were just a material supplier and intravenous securer.’ (Firehiwot, MUM).

It was believed, especially among the MUMs, that most birth and labour complications could be avoided if midwives were well utilised and team members were supported with professional oversight ensuring that staff followed clinical protocols. However, it was identified that the lack of senior medical doctors, who could provide clinical supervision, resulted in junior medical doctors or medical interns being responsible for managing the birth and associated complications. This increased the risk of birth complications or even death. Therefore, the MUMs believed that greater accessibility to senior medical doctors during childbirth would support midwives during complications.

‘It is good if senior medical experts are involved during the (birth of women) … In some cases, women (experience) haemorrhage after birth because, before the uterus contracts and returns to its place, they (residents) transfer the woman to the bed from the birth coach. There, she gets a haemorrhage.’ (Firehiwot, MUM).

While women in labour had been admitted to these birthing facilities, providing appropriate care was delayed due to the lack of senior medical personnel coupled with the inability of midwives to work to their full scope of practice, potentially increasing the risk of birth complications.

#### Communication Issues between Healthcare Providers

Team coordination and communication between teams during the management of PPPH were considered inadequate and ineffective by respondents in the current study. Descriptions of sluggish responses, especially between departments, could delay the effective management of PPPH. These communication delays often occurred between the birth unit and other hospital departments, usually laboratory and anaesthesia. One obstetrician felt that this prevented efficient medical care to women with PPPH, as he explained how the results from blood collections were often delayed in the hospital:

‘Staff take a long time to collect the blood sample for cross-match and (send back the result). Sometimes, the anaesthesia personnel may not be (in the birth unit) at the time (of PPPH’s occurrence) and (they) take time (to respond to the call for help).’ (Yihun, Obstetrician).

This study’s participants noted the importance of working as a team to effectively manage PPPH emergencies. Some midwives found the allocation of different roles to the clinicians during the PPPH management inefficient because communication was often ineffective in the team, as one expressed during an FGD:

‘… the team was not working … by dividing tasks … you could become confused … you did not know who was doing what.’ (Alemu, Midwife).

Participants recognised the importance of various healthcare professionals’ involvement in assessing, treating, stabilising, and initiating definitive management of women experiencing a PPPH. One participating obstetrician clarified how the clinical staff responded to a call for help and assisted with PPPH management:

‘Normally, when we make a call for help, many staff members respond and help in the management of PPPH. Without team (assistance), we cannot manage PPPH timely.’ (Yitayeh, Obstetrician).

The possibility of maternal death resulting from the poor management of PPPH can be minimised if the healthcare team has a well-formed leadership. Despite indicating they were aware of the advantages of team leadership, it was clear that many participants had experienced leadership challenges, often resulting in poor communication. Occasionally, multiple clinicians unknowingly performed the same activity, thus wasting time due to duplication. As one MUM explained:

‘Teamwork has to be improved. There has to be a leader who assigns tasks to other team members during the management of PPPH. Otherwise, you could see two professionals run to bring the same thing.’ (Ferede, MUM).

There were some disputes between the participants about the management of the women, which appeared to be dependent on the clinician’s profession. Ultimately, any conflict often resulted in a delay in the women receiving appropriate care. One obstetrician stated that such disputes hindered the provision of timely medical care:

‘… anaesthesia personnel preferred to give spinal anaesthesia for severe bleeding women, while obstetricians want to provide general anaesthesia. These arguments could take time.’ (Eshte, Obstetrician).

Another obstetrician supported this assertion:

‘… in the teamwork, some anaesthetists were reluctant to give general anaesthesia.’ (Eylgn, Obstetrician).

#### Systemic issues

Study participants expressed concern about the lack of support given by management to staff during emergencies. They revealed that during and after a PPPH emergency, there was a lack of hospital management presence with little to no debriefing about any challenges experienced. They felt the hospital management did not enquire about the challenges of the emergencies and whether any improvements could be made. The participants explained that supplies were frequently missing or limited, yet hospital management was disinterested in making improvements. In addition to the effective management of PPPH being hindered, the staff felt unsupported. As one MUM expressed:

‘… lack of support (from the hospital management) makes me hate the job …, they did not (provide) support and supervision.’ (Ferede, MUM).

Participants further explained that hospital management refused to allow the preparation of an emergency kit to manage PPPH. As the kit would contain essential fluids and medications necessary to manage PPPH, staff could save precious minutes during an emergency, hopefully reducing poor outcomes due to complications. As explained by one MUM:

‘… there is no understanding of the difficulty of managing PPPH (among the management staff of the hospital). For example, when we took drugs and intravenous fluid from the pharmacy to prepare an emergency kit in the birth unit, management staff told midwives why such preparation was needed in the presence of a nearby pharmacy. So, they think that organising these items in the birth unit is pointless…’ (Firehiwot, MUM).

#### Education, Training and Resourcing Issues

There were also concerns about the training of clinicians. One MUM stated that ‘almost half of the midwives were trained’ in the management of PPPH (Firehiwot, MUM). Contrasting views, however, suggested that clinicians did not receive adequate training. Due to the lack of training, there is concern about recognition of the onset of PPPH causing a delay in the medical care of PPPH. One MUM mentioned that staff did not receive in-service training:

‘… most of the midwives did not have direct training related to the management of PPPH … they came directly from a training school.’ (Ferde, MUM).

The findings showed that even those midwives who have worked in the profession for 8 years felt poorly educated in the management of PPPH:

‘I have been working for 8 years in a career, but I did not get any training all these years.’ (Mulualem, Midwife).

The participants further expressed concerns about the lack of the necessary medicine and resources to perform their roles during emergencies properly. Most participants agreed there was a lack of the necessary uterotonic drugs for the management of PPPH, which they believed contributed to the delay in receiving the appropriate care. As one midwife stated:

‘… uterotonic drugs should always be available, but they were not; there is a lack of supplies of this drug.’ (Miskir, Midwife).

Another midwife stated:

‘… no (uterotonic) drugs to be used for (the management of PPPH most of the time).’ (Asmare, Midwife).

Whilst acknowledging the shortage of uterotonic drugs compromised the management of PPPH, some study participants were optimistic. They described the presence of a nearby pharmacy as supportive, believing that the hospital pharmacy close to the birth unit provided maternal healthcare providers quick access to the necessary drugs to manage PPPH. In turn, they believed this timely access to the necessary medication contributed substantially to the successful management of PPPH. One MUM said that:

‘The birth unit had … a near (by) pharmacy where we would get drugs for PPPH, and this benefited many women.’ (Firehiwot, MUM).

Similarly, another MUM reported that:

‘There are uterotonic drugs …’ (for a woman who develops) ‘complications’ (during birthing and healthcare providers had provided drugs) ‘… right away’ (for women experiencing PPPH) (Ferede, MUM).

Participants also identified a shortage of blood, which contributed to the delay in providing appropriate care to women experiencing PPPH. Lack of whole blood limits maternal clinicians’ ability to offer lifesaving treatment for bleeding complications following birth. One MUM stated:

‘… some of the blood types may not be available, for example, O.’ (Alemu, MUM).

Another midwife stated:

‘Blood rarely saw, especially O negative blood type.’ (Mengistu, Midwife).

Several study participants said that the lack of uninterrupted and steady essential resources, including autoclaves, power supplies, and oxygen were barriers to PPPH management.

A lack of constant power supply was of particular concern in the operating theatre, where ventilators, anaesthesia monitors, and surgical equipment could fail to operate, jeopardising those women already severely compromised. One study participant was frustrated and stated:

‘… sometimes you may not get adequate light during operation in the theatre, which … frustrated me.’ (Eshte, Obstetrician).

Another concern for some participants was the lack of access to oxygen during emergencies. Midwives were especially concerned that oxygen was portable, as there was often an unnecessary wait while oxygen was brought from another hospital department. As one study participant noted:

‘It was challenging to get oxygen … in the ward …, and you have to wait until (someone) brought oxygen to the labour ward.’ (Mekdes, Midwife).

With these challenges in accessing essential resources, many participants were concerned that effective management of PPPH was often delayed, leading to unnecessary poor outcomes.

The participants recognised that planning is necessary to provide adequate medical responses for birthing emergencies. They understood that preparation and planning were key for better management of PPPH. More precisely, they viewed the staff’s awareness, preparation, and follow-up of women for any sign of PPPH as areas that needed improvement in these hospitals. As one MUM said:

‘… necessary supplies for the management of PPPH needs preparation in advance … Midwives should always be conscious and strictly make a follow up for every single woman following birth … for the occurrences of PPPH. It can happen any time.’ (Firehiwot, MUM).

Similarly, one midwife reaffirmed the need to prepare an emergency kit to manage PPPH. During the FGDs, he commented:

‘The birth unit must prepare for emergency medical care … always ready and alert.’ (Desta, Midwife).

Contrasting views from MUMs indicated, however, that there were some preparations in the birth units to manage PPPH. This included ensuring that the required equipment, medications, and other supplies for the treatment of PPPH were available. This practice was perceived as an essential responsibility of a MUM:

‘In the course of the management, my everyday main role was to check if there were (supplies and materials within the prepared kit) needed for the management. It is because sometimes the employee had taken out materials and supplies to use for non-emergency cases.’ (Ferede, MUM).

These findings suggest that midwives, MUMs, and obstetricians lack consensus about the preparation for PPPH management in these hospitals.

#### Lack of identification and referral

The participants identified the importance of screening women during antenatal care to identify those at risk of PPPH and then linking them to a referral hospital for definitive management. However, they also acknowledged that this would reduce the number of women having a PPPH, not eliminate the risk. One MUM discussed the need to refer high-risk women to tertiary referral hospitals from lower healthcare centres, which could only happen with the proper screening:

‘If there is an identification of a woman who is at (risk) of PPPH during antenatal care, … she should be referred to tertiary referral hospital as soon as the labour starts. Women should be informed about the need of going to the referral hospital immediately when labour begins.’ (Alemu, MUM).

## Discussion

This was the first qualitative study conducted in northwest Ethiopia, exploring the views and experiences of maternity healthcare professionals about the barriers to the management of PPPH following in-hospital births. The delay in access to appropriate and quality care was linked to numerous issues that hindered the management of PPPH following in-hospital births in northwest Ethiopia. The barriers discussed by the maternity healthcare providers were many and varied issues, including ‘workforce’, ‘communication between healthcare providers’, ‘professional conflict’, ‘failure of management systems’, ‘education and training’, ‘resourcing’, ‘lack of preparation’, and ‘lack of identification and referral’.

This study’s findings revealed that midwives were underutilised in these hospitals. Midwives appeared to lack job descriptions and potentially were not working at their full scope of practice, resulting in inefficient work practice as they roamed the hospital, waiting for instructions from the medical doctors. The ineffective use of midwives may have exposed other caregivers to a heavier workload. This issue can delay the care provided to women, increasing the risk of developing childbirth complications, such as PPPH. Allowing midwives to practice to their full scope or enhancing their role during the management of PPPH, especially by providing them with a job description, would improve the women’s care and reduce the risk of developing PPPH following in-hospital births [26]. In addition, providing frequent training opportunities for all clinical personnel, especially junior staff, is essential. Earlier research has also revealed that multidisciplinary team training for birthing emergencies often improves teamwork, leading to better outcomes [7].

Findings in this study also revealed that the maternity healthcare providers were concerned that the coordination and communication between teams were inadequate, leading to ineffective management of PPPH. Most commonly, there was a delay in the response from the anaesthesia department when called to emergencies. This finding suggests that there was a delay in dealing with life-threatening bleeding linked to labour and childbirth. Delaying management to such a case could be fatal because the women’s condition could decline much quicker than expected, making it impossible to perform lifesaving intervention. As a result, women, particularly those with uncontrollable PPPH, died due to management delays in these hospitals. Therefore, there is an urgent need to improve the responses to the call for help. This can be achieved by regularly assigning medical staff from the anaesthetic department to the birth unit. Although this will not address this issue, it could provide safe maternity care without delay, which is ideal for preventing maternal mortality and morbidity [27].

This study’s findings suggested that the hospitals were not providing supportive supervision, such as regular visits to the birth unit and initiatives to consult clinical personnel about their challenges in managing PPPH. Similar findings were also reported in Tanzania [28]. Along with other problems, a lack of supportive supervision could reduce motivation among maternal healthcare providers [29, 30], resulting in poor morale [31]. Staff burnout is often associated with poor morale, causing staff attrition and fewer healthcare workers working in low-resource settings. This becomes a perpetual cycle that eventually affects the hospital’s healthcare services. To stop this cycle, more supervisory support from the hospital’s management body is needed for their maternal healthcare providers to foster a positive working environment in the future.

The maternity healthcare staff reported that the hospital’s management body could not understand the need to prepare a PPPH emergency kit in case emergency essential supplies and materials were needed for better management of labour and birth complications. Linked to this lack of preparation, a delay of care could result in morbidity of PPPH, for example, in the case of birth trauma, significant blood loss could be prevented by timely repairing. These hospitals appeared unprepared for emergencies, including the management of PPPH. A clinical protocol needs to be implemented based on the current evidence in these hospitals. This would standardise the preparation required to provide safe maternal healthcare at the right time. Also, improving communication between the hospital management body and the clinical staff is needed. The birth unit’s emergency healthcare team could include non-clinical staff from the management body to improve communication and information exchange about the required preparation and resources in these hospitals.

Similar to research from Ghana [32], this study found that obstetricians and anaesthetists frequently disagreed about what care should be delivered to women based on their specialities. Although the conflict between providers can delay care and hamper efficient management of birthing emergencies, it is not uncommon due to individual variations, communication gaps, and other organisational issues [32]. Hence, the development and implementation of relevant hospital policies using current evidence-based practice should standardise care and assist in the healthy resolution of disagreements. In this study, the participants disclosed that there were disputes over the route of administering some medication in the operating room, which could be resolved effectively by implementing well-defined standards for a medication administration policy based on best practice [6].

The study of maternal healthcare providers in the participant hospitals revealed that midwives, MUMs, and obstetricians report that they do not receive adequate training about PPPH. This finding confirms similar reports by Beltman et al. [33] and Geleto et al. [34] in Africa, who stated that many healthcare staff did not have adequate training in this setting. The evidence suggests that due to a shortage of qualified midwives, assessing and determining the exact cause of labour complications for definitive management and thus reducing the risk of developing PPPH can be challenging [34]. Providing in-service training for maternal healthcare providers and adequate supplies is important.

Whilst the concerns about workforce shortages are seen in healthcare globally, the study participants had genuine concerns about poor planning for emergencies and the lack of available resources necessary when PPPH occurs. Many healthcare workers share this experience in other African nations [34, 35], where there is an undersupply of medications, inefficient oxygen access, and poor power supplies and autoclaving. As these tertiary referral hospitals in Ethiopia care for a high number of pregnant women with surgical and medical conditions, they may encounter a shortage of resources. To provide safe and sustainable care that ensures the best outcomes for women, hospitals must be well facilitated with adequate resources, including reliable infrastructure for emergency care. Perhaps one strategy could be to secure stable funding to avoid disruptions. Hospitals collaborating with local and international donors could ensure financial support for hospital infrastructures and supplies such as uterotonic drugs. This would enable these hospitals to provide safe maternity healthcare services and consequently reduce women’s risks of morbidity and mortality from PPPH after in-hospital births.

Like other African studies [33, 34], the study’s participants revealed they do not receive adequate education about PPPH management and felt unprepared for these emergencies. Considering Ethiopia’s MMR is high compared to high-resource countries, perhaps successful strategies used to prepare the staff for emergencies should be implemented. To improve the clinical management and communication between health professionals, ongoing education focused on managing obstetric emergencies is frequently undertaken by maternity care clinicians in high-resource countries with good outcomes [36]. Introducing regular simulations and education enables the whole maternity healthcare team to work together to improve clinical management and team communication in emergencies. Improving clinical competence through training may improve the maternity health professional’s confidence and encourage them to work within their scope of practice, especially the midwives. The midwives would have the confidence to independently assess the women to determine the exact cause of labour complications, thus implementing definitive management and reducing the risk of developing PPPH. In turn, improving maternity health professionals’ clinical competence may improve their confidence in their skills, and potentially improve staff morale, thus reducing staff attrition. This study has a limitation. The data were translated from one language to another, and although checked by two bilingual researcher nurses, there is a risk that some misinterpretation may have occurred during translation.

## Conclusion

This study identified multiple modifiable barriers to the management of PPPH in northwest Ethiopia. The findings reflect the concepts found in one aspect of the Three Delays model – the delay in receiving appropriate and quality care. Maternal healthcare providers identified many barriers that prohibited access to appropriate and quality care for women experiencing PPPH following in-hospital births. The identified workforce issues include staff not being supported to practice to their full scope; poor communication between existing staff; poor preparation; and insufficient resources, especially power supply, medication, and oxygen. A detailed plan will introduce strategies to improve the delivery of maternity care, aiming to reduce the incidence of PPPH and lower its mortality and morbidity. Our suggested plan underpins three cornerstones of evidence-based care—education, preparation for emergencies, and resourcing. First, the skill mixes of senior and junior staff providing care must be appropriate. All staff, including midwives, should be able to practice to their scope. Junior staff should have supportive supervision. Hospital management and staff should have ongoing education to ensure they are cognisant of recent practice. Second, hospital management and staff need to be prepared for emergencies. Staff need to be able to access resources such as PPPH emergency kits prepared for emergencies. Finally, communication between health professions needs to be improved through training. Regular team-based obstetric emergency training has been shown to improve communication between healthcare professions. Ensuring that all staff are involved in implementing policies as recommended by WHO [37] will enable them to become familiar with the latest evidence. Hospital management also needs to support the implementation of a clinical protocol based on the current evidence in these hospitals [37].

## Electronic supplementary material

Below is the link to the electronic supplementary material.

Supplementary Material 1

## Data Availability

Although the researcher cannot submit data to a repository due to prior agreement with the study participants, the datasets used and/or analysed during the current study are available from the corresponding author on reasonable request.
