# Supplementary material for: Barriers to effective management of primary postpartum haemorrhage following in-hospital births in northwest Ethiopia: healthcare providers’ views using a qualitative approach
Source: BMC Pregnancy Childbirth. 2022 Oct 8;22:755. doi: 10.1186/s12884-022-05071-6 (PMC9548148; doi:10.1186/s12884-022-05071-6)
Supplement: Supplementary file 1 — Supplementary Material 1 [file 12884_2022_5071_MOESM1_ESM.docx]

Focus Group Discussion Tool

Midwives

Focus group discussion ………………. Duration: ………. to ……….

Role of Facilitator

Your transcribed words are not included in the analysis

You are a receiver, not a transmitter.

Instructions for Facilitator

Refer to the timestamps on the guide to help you stay on track

Ensure that the participants are holding the audio recorder whenever possible

Welcome and introductions

Collect signed consent forms

Remind the participant to speak into the audio recorder as much as possible.

Note: Ensure that participants have had a chance to remember the number of births in the hospital in the last 12 months by saying, ‘I would like to ask you to remember the number of women who received management for PPPH following in-hospital births in the last 12 months.’ Pause for a moment.

1) Please tell me about your experiences managing a PPPH in the health service.

2) Describe the most important activities that were done very well.

Probes

Can you tell me about the preparation of the emergency equipment, for example, the PPPH emergency kit to manage the care of a woman experiencing PPPH?

Can you tell me about your training for the management of a woman experiencing PPPH?

Can you tell me how you communicate with other staff during the night shift to manage a woman experiencing PPPH?

3) Describe the challenges.

Probes

What are some of the challenges of the system for the management of women experiencing PPPH? (e.g., supplies, refrigeration to store oxytocin, blood, distribution of supply, motivation of healthcare workers, etc.).

What are some of the challenges at the level of the healthcare provider? (e.g., adherence to guidelines and recommendations, immediate management for an obstetric emergency, access to expert’s care, motivation of healthcare workers, knowledge and skills for certain procedures vital to the management of a woman experiencing PPPH, knowledge exchange opportunities between senior and junior healthcare providers, etc.).

4) Describe the support you had.

Probes

What are some of the system supports for the management of women experiencing PPPH? (e.g., readily available supply, availability of protocols for the management of a woman experiencing PPPH, simulation exercises related to the management of a woman experiencing PPPH, onsite/bedside training and feedback to aid healthcare workers in strategies related to the management of a woman experiencing PPPH, training related to the management of a woman experiencing PPPH, etc.).

5) Describe how the team, or teams, communicated with each other.

Probes

Can you tell me how the team/s communicated with each other to manage a woman experiencing PPPH?

Can you tell me about your overall comment on the performance of the team/s during the management of a woman experiencing PPPH?

Can you tell me who is responsible for leading the team during the management of a woman experiencing PPPH?

6) Do you have any additional information that you would like to share about the management of PPPH?

Thank participants and wrap up.

Midwife Unit Manager

Interview ………………. Duration: ………. to ……….

Role of Facilitator

Your transcribed words are not included in the analysis

You are a receiver, not a transmitter.

Instructions for Facilitator

Refer to the timestamps on the guide to help you stay on track

Ensure that the interviewee is holding the audio recorder whenever possible

Welcome and introductions

Collect signed consent forms

Remind the interviewee to speak into the audio recorder as much as possible.

Note: Ensure that interviewees have had a chance to remember the number of births in the hospital in the last 12 months by saying, ‘I would like to ask you to remember the number of women who received management for PPPH following in-hospital births in the last 12 months.’ Pause for a moment.

1) Please tell me about your experiences managing a PPPH in the health service.

2) Describe the most important activities that were done very well.

Probes

Can you tell me about the preparation of the emergency equipment, for example, the PPPH emergency kit to manage a woman experiencing PPPH?

Can you tell me about your training for the management of a woman experiencing PPPH?

Can you tell me how you communicate with other staff during the night shift to manage a woman experiencing PPPH?

3) Describe the challenges.

Probes

What are some of the challenges of the system for the management of women experiencing PPPH? (e.g., supplies, refrigeration to store oxytocin, blood, distribution of supply, and motivation of healthcare workers, etc.).

What are some of the challenges at the level of the healthcare provider? (e.g., adherence to guidelines and recommendations, immediate management for an obstetric emergency, access to expert’s care, motivation of healthcare workers, knowledge and skills for certain procedures vital to the management of a woman experiencing PPPH, knowledge exchange opportunities between senior and junior healthcare providers, etc.).

4) Describe the support you had.

Probes

What are some of the system supports for the management of women experiencing PPPH? (e.g., readily available supply, availability of protocols for the management of a woman experiencing PPPH, simulation exercises related to the management of a woman experiencing PPPH, onsite/bedside training and feedback to aid healthcare workers in strategies related to the management of a woman experiencing PPPH, training related to the management of a woman experiencing PPPH, etc.).

5) Describe how the team, or teams, communicated with each other.

Probes

Can you tell me how the team/s communicated with each other to manage a woman experiencing PPPH?

Can you tell me about your overall comment on the performance of the team/s during the management of a woman experiencing PPPH?

Can you tell me who is responsible for leading the team during the management of a woman experiencing PPPH?

6) Do you have any additional information that you would like to share about the management of PPPH?

Thank participants and wrap up.
